# A LIFE COURSE PERSPECTIVE ON FACTORS UNDERLYING NURSING HOME STAFF RETENTION

**DOI:** 10.1093/geroni/igae098.4100

**Published:** 2024-12-31

**Authors:** Waqar Ahmad, Jennifer Morgan, Elisabeth Burgess, Eric Wright

**Affiliations:** Georgia State University, Atlanta, Georgia, United States; Georgia State University, Atlanta, Georgia, United States; Georgia State University, Atlanta, Georgia, United States; Georgia State University, Atlanta, Georgia, United States

## Abstract

Nursing homes continue to experience persistent challenges in retaining nursing home staff. Understanding the life course context of nursing home staff has the potential to shed needed light on this persistent issue. The life course perspective emphasizes understanding the dynamic interplay of personal, social, organizational, and historical factors and how this dynamism influence lives. Using cross-sectional survey data from 678 nursing home staff working in 23 nursing homes, we use ordinal logit regression to estimate a predictive model consisting of demographic, job quality and life course factors predicting employment intentions. Life course-relevant findings include: intergroup differences in employment intentions by life course markers and identities with career stage, benefits status, and breadwinner status demonstrating significant impact on intent to stay. Further, we find evidence that cumulative disadvantage, emotional burnout and other care roles have additional significant impacts on intent to stay. This study has practical implications for nursing home leadership and policy makers. Nursing home leaders need to amend their conventional strategies of retaining the LTC workforce by creating tailored supports for workers while simultaneously addressing job quality deficits. Access to mental health services, increased leave for caregiving and respite, improved access to high-quality health insurance and benefits that assist this workforce to deal with contingency factors and career mobility (e.g. small grants, tuition remission) have the potential to alleviate some of the instability in this often-vulnerable workforce.

